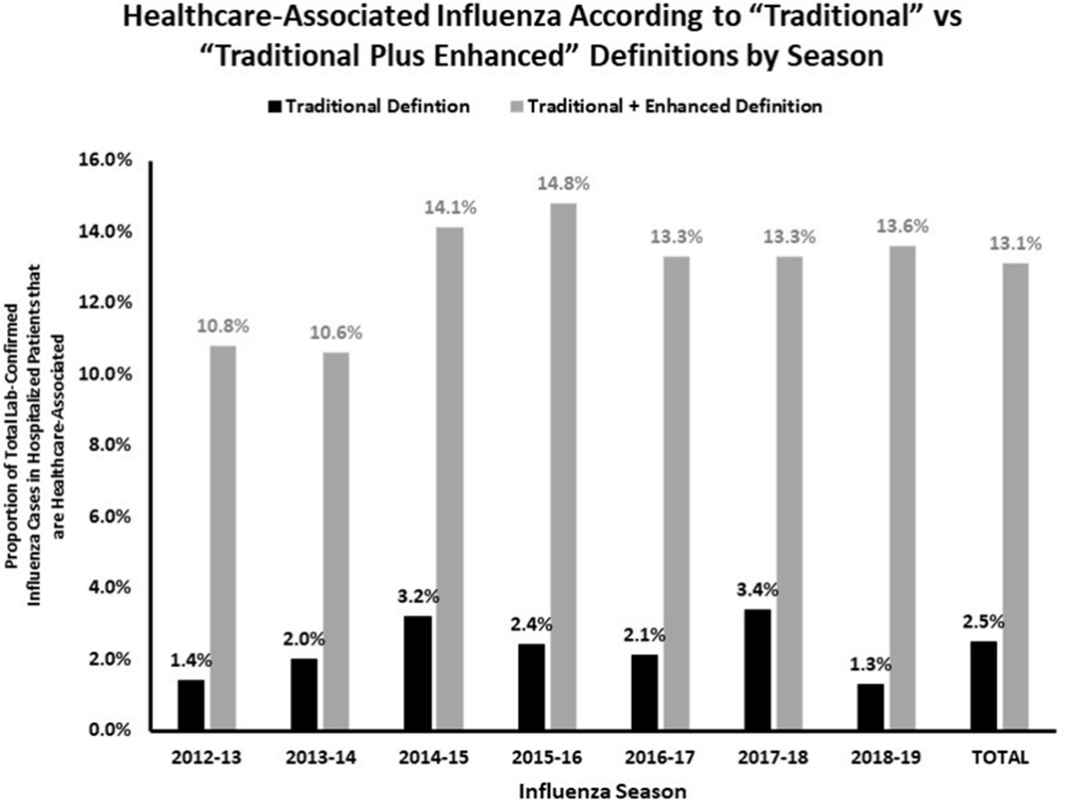# Traditional Definition of Healthcare-Associated Influenza Underestimates Cases Associated with Other Healthcare Exposures

**DOI:** 10.1017/ash.2021.23

**Published:** 2021-07-29

**Authors:** Erin Gettler, Thomas Talbot, H. Keipp Talbot, Bryan Harris, Danielle Ndi, Edward Mitchel, Tiffanie Markus, William Schaffner

## Abstract

**Background:** Healthcare-associated transmission of influenza leads to significant morbidity, mortality, and cost. Most studies classify healthcare-associated viral respiratory infections (HA-VRI) as those with a positive test result after the first 3 days following admission, which does not account for healthcare exposures prior to admission. Utilizing an expanded definition of healthcare-associated influenza, we aimed to improve the estimates of disease prevalence on a population level. **Methods:** This study included laboratory-confirmed cases of influenza in adult and pediatric patients admitted to any acute-care hospital in a catchment area of 8 counties Tennessee identified between October 1, 2012, and April 30, 2019. Surveillance information was abstracted from hospital and state laboratory databases, hospital infection control practitioner databases, reportable condition databases, and electronic health records as a part of the Influenza Hospitalization Surveillance Network (FluSurv-NET) by the Centers for Disease Control and Prevention (CDC) Emerging Infections Program (EIP). Cases were defined as healthcare-associated influenza laboratory confirmation of infection occurred (1) on or after hospital day 4 (“traditional definition”), or (2) between hospital days 0 and 3 in patients transferred from a chronic care facility or with a recent discharge from another acute-care facility in the 7 days preceding the current index admission (ie, enhanced definition). The proportion of laboratory-confirmed influenza designated as HA-VRI using both the traditional definition as well as with the added enhanced definition were compared. Data were imported into Stata software for analysis. **Results:** We identified 5,904 cases of laboratory-confirmed influenza in hospitalized patients over the study period. Using the traditional definition for HA-VRI, only 147 (2.5%, seasonal range 1.3%–3.4%) were deemed healthcare associated (Figure 1). Adding the cases identified using the enhanced definition, an additional 317 (5.4%, range 2.3%–6.7%) cases were noted in patients transferred from a chronic care facility for the current acute-care admission and 336 cases (5.7%; range, 4.1%–7.4%) were noted in patients with a prior acute-care facility admission in the preceding 7 days. Using our expanded definition, the total proportion of healthcare-associated influenza in this cohort was 772 of 5,904 (13.1%; range, 10.6%–14.8%). **Conclusion:** HA-VRI due to influenza is an underrecognized infection in hospitalized patients. Limiting surveillance assessment of this important outcome to just those patients with a positive influenza test after hospital day 3 captured only 19% of possible healthcare-associated influenza infections across 7 influenza seasons. These results suggest that the traditionally used definitions of healthcare-associated influenza underestimate the true burden of cases.

**Funding:** No

**Disclosures:** None

**Figure 1.**